# Palliative Care Advocacy: Why Does It Matter?

**DOI:** 10.1089/jpm.2019.0696

**Published:** 2020-07-15

**Authors:** Katherine Irene Pettus, Liliana de Lima

**Affiliations:** International Association for Hospice and Palliative Care (IAHPC), Houston, Texas, USA.

**Keywords:** advocacy, palliative care, policy and budget, primary health care, United Nations system

## Abstract

Evidence-based advocacy within the United Nations system for integration of palliative care into primary health care is essential to inspire and nurture the political will necessary to support the development and funding of national palliative care policy. National policy is, in turn, essential to underwrite clinical delivery that leaves no patient behind. Although International Association for Hospice and Palliative Care (IAHPC) has engaged in advocacy since its inception, the board decision to prioritize advocacy as part of the organization's strategic plan has taken it to a more formal level. This piece summarizes the content of the basic advocacy course released for IAHPC members, defines palliative care and advocacy, distinguishes advocacy from lobbying, discusses how an international organization such as the IAHPC advocates for palliative care at the global level, and clarifies the vital feedback loop between advocacy and clinical practice.

## Introduction

In 2019, the board and staff of the International Association for Hospice and Palliative Care (IAHPC), comprising a global multidisciplinary cross section of experts, developed a five-year strategic plan for 2020–2024.^1^ They identified four mutually reinforcing areas of work, one of which was advocacy. Although IAHPC has engaged in advocacy since its inception, this board decision prioritized it within the organization's strategic plan. This piece summarizes the content of the basic advocacy course released for IAHPC members in 2019. It defines palliative care and advocacy, distinguishes advocacy from lobbying, discusses how an international organization such as the IAHPC advocates for palliative care at the global level, and clarifies the relationship between advocacy and clinical practice.

## Why This Is Important?

The basic assumptions comprising the IAHPC advocacy theory of change are that (1) palliative care is an element of the right to health, *not a privilege*, and that (2) since rights, by definition, are universal and publicly guaranteed,^2^ implementing them entails the development of public *policy.* We recognize that appropriate clinical palliative care is important, and support training of professionals and caregivers. However, to ensure that no person is left behind, we must all advocate for the inclusion of palliative care in policies and norms. Our membership and palliative care partners can (and should) ask their governments to implement the provisions now extant in international law so as to ensure that *national* laws and regulations include delivery of palliative care.

## Advocacy

The noun “ad*voc*acy” and verb “to advocate” are derived from the Latin “*voc,*” which is related to “speech” and implies “speaking for others.”^3^ Etymologically, the term “advocate” has been associated with courtrooms and judicial processes. Advocacy for health policy is defined as “the processes by which the actions of individuals or groups attempt to bring about social and/or organization change on behalf of a particular health goal, program, interest, or population. Health advocacy includes educating policymakers and the public about evidence-based policy.”^4^ Palliative care advocacy is strongest when it includes the *voices* of direct stakeholders, those who deliver and receive essential services, and can *testify, witness* to the vast unmet need for palliative care. The IAHPC advocacy program partners with other palliative care organizations committed to including the *voices* of direct stakeholders.^5^ Our policy experts testify to the benefits of palliative care services for health systems in general, and for patient and caregiver quality of life in particular.

Its foundation in speech means that effective palliative care advocacy relies on a shared conceptual framework and vocabulary. At the multilateral level (where organizations of member states develop and oversee policy), this is the human rights framework, which has been evolving since the post-World War 2 era. As a result of concerted advocacy, the right to palliative care as a component of the right to the highest attainable standard of physical and mental health is now embedded in human rights **s**tandards articulated by the Special Rapporteurs on the Right to Health, to be free from Torture, Cruel, Inhumane Treatment, and reports of the Independent Expert on the Rights of Older Persons.^6^

The IAHPC has official status to advocate in these multilateral meetings of United Nations (UN) treaty bodies and to press human rights standards regarding palliative care because it is accredited as a “nongovernmental (civil society) organization” in consultative status with the UN's Economic and Social Council (ECOSOC), and as a Non-State Actor (NSA), with the World Health Organization (WHO). Our advocacy for palliative care entails regular funded participation at meetings of UN treaty bodies such as the WHO, the Commission on Narcotic Drugs, and the Human Rights Council. These treaty bodies regularly issue consensus-based declarations, resolutions, and reports on public health, “drug control,” sustainable development, aging, and related topics, all of which, if appropriate, should now include references to palliative care and internationally controlled essential medicines.

## International Conventions

International conventions are treaties or agreements between countries regarding issues that require a coordinated approach. The term “international convention” is often used interchangeably with “international treaty” and “international agreement,” and the whole collection of treaties is referred to as the International Policy Framework.

Conventions may be general or specific and involve two or more member states. Conventions between two member states are called bilateral treaties; conventions between a small number of states (but more than two) are called plurilateral treaties; and conventions between a large number of states are called multilateral treaties. Conventions and treaties signed by member states bind them to the terms of that agreement through the domestic application of the treaty provisions.

Multilateral conventions such as the Single Convention on Narcotic Drugs^7^ are supervised by multilateral “treaty bodies” such as the Commission on Narcotic Drugs, which convenes twice a year in Vienna, Austria. The IAHPC has official status to participate in those meetings and advocates that member states improve access to internationally controlled essential medicines, per their “treaty obligations” under the Single Convention and other “drug control” agreements.

As a result of coordinated advocacy, most of the documents approved by these treaty bodies, which are both normative and technical, now include the words “palliative care” as an essential component of primary health care,^8^ Universal Health Coverage, and by extension, sustainable development.^9,10^ Regional and national palliative care associations can leverage the multilateral policy commitments in these documents, which have been approved by all their respective governments, to advance service delivery at home. Effective advocacy activates a feedback loop that extends from member state treaty bodies and official documents, through civil society organizations, to the bedside and back again, as member states report their progress at global meetings.

The public health dimensions of the right to palliative care, and the obligations of the duty bearers (member states) are spelled out in World Health Assembly (WHA) resolution, “Strengthening of Palliative Care as a Component of Comprehensive Care throughout the Life Course.”^11^ This resolution, approved by all WHO member states in 2015, was informed by extensive consultation between the WHO Secretariat, member states, national, regional, and international palliative care organizations, and Human Rights Watch. Regional support for the right to palliative care is articulated in the African Union Common Position on Access to Controlled Substances,^12^ the Protocol on Rights of Older Persons,^13^ and the Organization of American States' Inter-American Convention on Rights of Older Persons.^14^

## Defining Palliative Care

A plethora of outdated and diverse definitions of palliative care has weakened the shared conceptual framework necessary for effective public health advocacy. In 2018, in an effort to overcome that challenge, the IAHPC organized a rigorous and inclusive global process whose objective was to produce a consensus-based definition. The process concluded with a definition of “palliative care” as “the active holistic care of individuals across all ages with serious health-related suffering due to severe illness and especially of those near the end of life. It aims to improve the quality of life of patients, their families and their caregivers.”^15^ Because service delivery of a universal right (on the ground, or at the bedside) entails the development and implementation of funded public policy, the definition stipulates that governments *must*

(1) adopt adequate policies and norms that include palliative care in health laws, national health programs, and national health budgets;
(2) ensure that insurance plans integrate palliative care as a component of programs;
(3) ensure access to essential medicines and technologies for pain relief and palliative care, including pediatric formulations;
(4) ensure that palliative care is part of all health services (from community health-based programs to hospitals), that everyone is assessed, and that all staff can provide basic palliative care with specialist teams available for referral and consultation;
(5) ensure access to adequate palliative care for vulnerable groups, including children and older persons; and
(6) engage with universities, academia, and teaching hospitals to include palliative care research as well as palliative care training as an integral component of ongoing education, including basic, intermediate, specialist, and continuing education.

The task of familiarizing governments with these recommendations, all of which align with the extant multilateral human rights framework already referenced, entails local, global, and regional advocacy for all six components of the consensus definition.

## Advocacy versus Lobbying

The distinction between advocacy and lobbying is an important one: health *advocacy* includes educating policymakers and the public about evidence-based policy, whereas *lobbying* includes “attempts to influence a legislative body through communication with a member or employee of a legislative body, or with a government official who participates in formulating legislation.”^16^ In many countries, awareness of this distinction between advocacy and lobbying can preserve the tax-free or nonprofit status of civil society organizations such as national palliative care associations. Some countries allow nongovernmental organizations (NGOs) to “lobby” for specific legislation, others do not. United States law limits NGOs lobbying activities to a minimum portion of the organization's overall budget and prohibits U.S.-based NGOs from engaging in lobbying activities in other countries. It does not, however, limit advocacy. Because the IAHPC is based in the United States, we are bound by U.S. law.

IAHPC *advocates* in the context of the UN system for national governments to comply with international law and human rights standards, but we are prohibited from *lobbying* for specific legislation in those countries. However, IAHPC can assist and support, through international advocacy and clinical education. We may engage in national advocacy only if invited to do so by legitimate national entities, and can never engage in lobbying. Depending on the regulatory framework of *their* countries, however, *national* associations may be allowed to do exactly that. IAHPC urges our advocacy partners in member palliative care associations to review their countries' regulatory frameworks regarding advocacy and lobbying restrictions and will provide guidance when requested.

## Other Challenges

Other challenges include limited government/public resources for health and social services, scarce philanthropic resources to train and fund the travel of regional and national advocates to global meetings, and a global health ideology that prioritizes reducing preventable mortality. Donor-funded palliative care advocacy requires deliverables such as verifiable policy change and measurable public health outcomes. Although palliative care advocacy efforts often bear fruit in the long term, quantifiable relationships between input and reportable results remain elusive. Because palliative care advocacy is complex and relationship based, its impacts tend to be indirect and nonlinear, making quantifiable evaluation challenging. Effective evaluation should be contextual and consider the theory of change underpinning the work.

Effective advocacy entails inspiring and nurturing political will, effective planning, and funded budgets. Nationally accredited NGOs, palliative care organizations, and professional associations, many of which are members of IAHPC, can make more headway in this challenging field by developing joint strategies.

## Conclusion

Widespread unfamiliarity with the palliative care approach to serious health-related suffering challenges palliative care advocacy at all levels. Misunderstandings are pervasive throughout the UN system, national health care systems, the media, and the general public. Policymakers and providers who are somewhat familiar with palliative care stigmatize this relatively new public health approach as *only* appropriate for end of life, or *only* for cancer, etc. Such misunderstandings undercut public policy and limit service delivery to the private and charitable sectors, which can only meet a fraction of the growing global need. Evidence-based testimony by providers trained in basic palliative care, and service users who have benefited, can support recognition of the universal nature of the right. Success stories can assuage uninformed concerns about additional costs of integrating palliative care services in the context of hospital-based systems and ally widespread fears that associate essential palliative care medicines with illegal “drugs.” The IAHPC Advocacy Program and the organization, its board, and staff members will continue to provide support and guidance to all our stakeholders, including global, regional, and national palliative care organizations, members, policy makers, patients, caregivers, and professionals working to relieve suffering and improve the quality of life of millions of patients around the globe.
